# Letter from the Editor in Chief

**DOI:** 10.19102/icrm.2026.17066

**Published:** 2026-06-15

**Authors:** Devi Nair


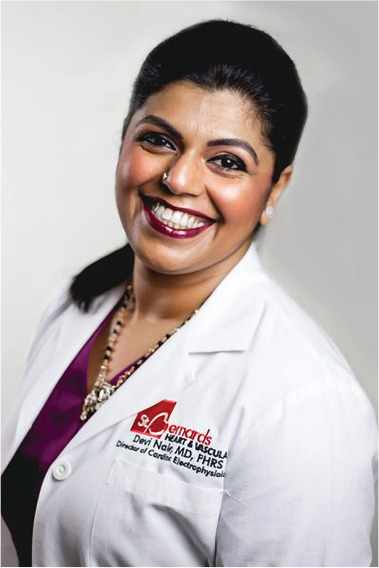


Dear Colleagues,

Welcome to the June 2026 issue of *The Journal of Innovations in Cardiac Rhythm Management*.

June is a month of thresholds. Across our training programs, the academic year turns over and, with it, a generation of physicians moves one step further into the field. Our graduating electrophysiology fellows are completing their final cases under supervision and preparing to stand on their own as independent operators. Our cardiology fellows who have matched into electrophysiology are trading the familiar rhythms of the general service for the unfamiliar grammar of the mapping system and the ablation catheter. Finally, a new class of internal medicine residents is arriving on the cardiology service for the first time, stethoscopes still stiff, learning to read the 12-lead electrocardiogram as a language rather than a puzzle. Three cohorts, three thresholds, and a single continuous lineage that binds the most junior resident to the most senior attending: the obligation to learn carefully; to be mentored generously; and, in time, to mentor in turn.

To those of you leaving fellowship for practice, a word of counsel from colleagues who have made the same passage. The transition from a program, where a more experienced operator is always within reach, to a laboratory in which the final judgment is yours is the steepest and most consequential of your career. Resist the temptation to measure your competence by the difficulty of the cases you can attempt; measure it instead by the clarity with which you know your own limits and by your willingness to ask for help when a case exceeds them. Cultivate the relationships that will outlast your training: the senior partner who will take your call at an awkward hour, the referring cardiologist who trusts your reasoning, and the industry engineer who knows your equipment better than you do. Also, keep one foot in the scholarly life. The habit of asking a precise question, gathering data honestly, and subjecting your own results to scrutiny does not belong to the academic alone; it is the discipline that keeps any clinician honest and the surest defense against the slow drift toward complacency that practice can encourage.

To our incoming electrophysiology fellows: you are entering a subspecialty that reinvents its tools faster than almost any other in medicine, and the temptation will be to chase each new platform before you have mastered the fundamentals it is built upon. Learn the anatomy and the electrophysiology first. The energy source will change; the conduction system will not. And, to the residents beginning cardiology this month: the careful re-reading of an electrocardiogram, the question asked at the bedside, the case that puzzles you and will not let you sleep—these are the raw material of every paper in this issue and of every advance our field has ever made. Write them down. The manuscripts that fill these pages began as exactly that kind of curiosity in someone not far ahead of you.

The work collected in this issue speaks, fittingly, to trainees at every stage. It spans an original comparison of two ablation strategies, a first-in-kind solution to a difficult device problem, a toxicologic emergency resolved at the bedside, a genetic diagnosis hidden in plain sight for decades, and a tracing that rewards the patient reader with one of the most extreme findings in the conduction-system literature.

In an original research contribution, Alnazeer, Fan, Giang, and colleagues^1^ compare robotic magnetic navigation against conventional manual catheter ablation for premature ventricular contractions in a single-center retrospective cohort of patients treated between 2019 and 2023. Among the per-protocol cohort of 86 patients, 67 undergoing robotic ablation and 19 undergoing manual ablation, the two approaches yielded comparable total procedure times, comparable fluoroscopy times, comparable 1-month symptom resolution, and comparable periprocedural complication rates, while class I/III anti-arrhythmic drug use fell substantially in both groups. The authors are admirably candid about what their data can and cannot support: because post-ablation premature ventricular contraction burden and follow-up ventricular function were not systematically captured, the findings are best read as hypothesis-generating and confined to procedural efficiency and early, largely subjective outcomes. For the trainee, the paper is as much a lesson in the honest framing of a retrospective single-center experience as it is a comparison of two technologies.

Sovari, Pourafkari, and Kim^2^ report what is, to their knowledge, the first off-label implantation of an Aveir™ AR (atrial) leadless pacemaker (Abbott, Chicago, IL, USA) in the right ventricle, undertaken in a 90-year-old woman with permanent atrial fibrillation, symptomatic bradycardia, severe functional tricuspid regurgitation, and a small right ventricular cavity. An initially implanted Aveir™ VR (ventricular) device produced recurrent loss of capture attributed to the small chamber and to interaction with the tricuspid valve; after extraction, the smaller-profile atrial device was deployed in the right ventricle and achieved stable fixation. The case is a clean illustration of the adaptability of modular leadless platforms and of a principle that applies far beyond this device: that anatomy, not habit, should dictate the choice of hardware, particularly in the elderly patient whose chambers and valves do not conform to the assumptions a system was designed around.

Chaudhry, Inanc, Khan, and colleagues^3^ describe a young man who presented unresponsive after an intentional polysubstance ingestion that included at least 600 mg of propafenone, with a sustained wide complex tachycardia, marked QRS prolongation, and severe metabolic acidosis. Within an hour of intravenous sodium bicarbonate and aggressive supportive care, the tachycardia resolved and the QRS narrowed, with continued normalization over the following day. The report is a vivid reminder that the surface electrocardiogram is not merely diagnostic but dynamic, that QRS duration can serve as a real-time gauge of class IC toxicity and of the response to treatment, and that a sodium channel poisoned by drug intake can often be rescued at the bedside before more-invasive measures are required.

Pande, Mukherjee, and Halder^4^ present a 57-year-old woman whose decades of transient loss of consciousness had been diagnosed and treated as epilepsy until recurrent torsades de pointes requiring direct-current cardioversion disclosed a markedly prolonged corrected QT interval. Genetic analysis identified a previously unreported, possibly pathogenic compound heterozygous variant in the *AKAP9* gene. With a dual-chamber implantable cardioverter-defibrillator, beta-blockade, mexiletine, and a higher base pacing rate, and with her anti-epileptic drugs discontinued, she remained free of arrhythmic events at 6 months, with an improved corrected QT interval. The case is a quiet argument for genotype-guided therapy and a cautionary tale about the diagnosis that hides for years behind a more familiar label.

Finally, in this month’s Tracing of the Month, Mondal, Muslim, Matia, and Dhua^5^ document a frail nonagenarian with an incessant wide-complex tachycardia of left bundle branch block morphology, transiently responsive to adenosine yet refractory to cardioversion and given to immediate reinitiation. Beneath the tachycardia lay a profoundly diseased infra-Hisian conduction system, with an H–V interval of 306 ms, among the longest documented in the literature, and a rare phenomenon of alternating bundle conduction during bradycardia. The tracing is exactly the kind of record that repays the patient reader and a fitting reminder to our newest trainees that the electrocardiogram and the intracardiac electrogram still have secrets to surrender to those willing to look closely.

If there is a thread that runs from the graduating fellow to the arriving resident and through every manuscript in this issue, it is that careful observation, honestly recorded and openly shared, is the engine of our discipline. I will close, then, with an invitation, and it is meant as much for the trainee as for the established investigator. If you have a case that taught you something, a tracing that puzzled you, a series that challenges a prevailing assumption, or a question worth a rigorous study, this journal exists to publish it. Bring your mentors into the work, give your trainees a place on the byline they have earned, and let the habit of scholarship travel with you into whatever practice you are entering. The pages of this journal are open to you.

I am grateful, as always, to our authors, reviewers, and editorial team for their work and to you, our readers, for your continued engagement with the journal. To those of you finishing training this season: congratulations, and welcome to the lifelong company of those who keep learning.

Warm regards,


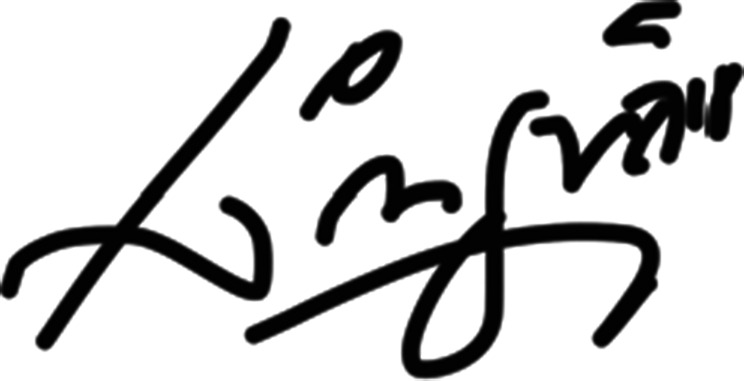


Dr. Devi Nair, md, facc, fhrs

Editor-in-Chief

*The Journal of Innovations in Cardiac Rhythm Management*

Director of the Cardiac Electrophysiology & Research,

St. Bernard’s Heart & Vascular Center, Jonesboro, AR, USA

White River Medical Center, Batesville, AR, USA

President/CEO, Arrhythmia Research Group

Clinical Adjunct Professor, University of Arkansas for Medical Sciences

Governor, Arkansas Chapter of the American College of Cardiology

drdgnair@gmail.com
